# Erie County Medical Society

**Published:** 1883-02

**Authors:** 


### ERIE COUNTY MEDICAL SOCIETY.

The annual meeting of the Erie County Medical Society was held at the college building on the 9th ult. The President, Dr. T. M. Johnson, in the Chair; A. M. Barker, Secretary.

The committee on membership reported in favor of the admission of Drs. A. A. Hubbell, C. Weil, G. Frank and George E. Fell.

The following new applications for membership were received and referred to the committee, F. H. Potter, J. N. Putnam, W. G. Gregory, E. H. Long, J. W. S. Hunter, J. H. Pryor.

Dr. E. Storck, chairman of the "Board of Censors," reported that the names of a number of persons practicing in violation of the law had been furnished the district attorney, but owing to a lack of sufficient proof the cases had not been, as yet, brought before the courts.

The Board were notified of a person in one of the country towns who was practicing illegally. They wrote him a letter in regard to the matter, and he at once had betaken himself to parts unknown.

Dr. F. F. Hoyer believed the proper place to try these cases was before a police justice. He said that conviction had been obtained in the police court in New York in several cases, and the violators fined.

Dr. J. B. Samo, Librarian, reported that during the year just closed the additions to the library were, a "Treatise on the Diagnosis and Treatment of Diseases of the Chest," by William Stokes, of Dublin, and a fasciculus, consisting of two large plates, of certain affections of the skin. Also, forty-four bound volumes of the *London Lancet*, these being a gift to the Erie County Medical Society, from Dr. John Boardman. These volumes date from the beginning of that valuable journal, in 1823, and extend to 1845, embracing a period of twenty-two years. The society is to be congratulated on the possession of so valuable a collection. A vote of thanks was extended to Dr. Boardman for the liberality and thoughtful kindness implied in his donation.

Dr. F. W. Abbott, Treasurer, presented his annual report. It showed that the society had expended one hundred dollars during the past year. This included fifty dollars legal expenses paid to A. G. Rice, attorney for the society.

The Secretary called the attention of the society to a blank book of certificates which had never been used. He suggested the propriety of purchasing a proper seal and furnishing each member with a certificate of membership.

He was directed to purchase such a seal and have a new list of the members printed.

The subject of the Code of Ethics, as revised by the New York State Medical Society, was discussed. The following resolution, offered by Dr. H. R. Hopkins at the semi-annual meeting in June, was taken from the table:

"*Resolved*, That this society approves of the recent action of the State Society in revising the Code of Ethics."

Dr. C. C. F. Gay moved, as a substitute for the above, that the "Erie County Medical Society disapproves the action of the State Medical Society, at their last meeting, in adopting the report of the special committee in revising the Code of Medical Ethics"

Dr. A. R. Davidson said that he was heartily in favor of Dr. Gay's resolution, but in his opinion it did not go far enough; he hoped that the sentiments of the society on this important question would be expressed in no uncertain way. The resolution offered left our delegates free to vote on this question as their individual opinions dictated. It was well known that a majority of the delegates had already expressed themselves in favor of the New Code. While willing to give them perfect liberty in the discussion of the subject before the State Society, he believed that they should receive the instructions of the society as to how they should vote. He therefore presented the following as an amendment to Dr. Gay's resolution:

WHEREAS, The Medical Society of the State of New York, at its last annual meeting, did adopt a New Code of Ethics, which provides that "Members of the Medical Society of the State of New York, and of the medical societies in affiliation therewith, may meet, in consultation, *legally* qualified practitioners;" and,

WHEREAS, The clause above quoted is in direct opposition to the Code of the American Medical Association, with which this society desires to retain its relations, and also opposed, in the opinion of a majority of the members of this society, to the dignity and true interests of scientific medicine, therefore,

*Resolved*, That this society regrets the action of the State Society in adopting a New Code of Ethics, and respectfully asks that the action may be reconsidered and reversed at the next annual meeting.

*Resolved*, That the delegates of this society be instructed to vote, on this subject, in accordance with these resolutions, and that an authenticated copy of the same be forwarded to the Secretary of the State Medical Society.

These resolutions called forth a lively discussion, participated in by a majority of those present, during which the following letter was read by the Secretary:

*To the President of the Erie County Medical Society:*

DEAR SIR—I regret very much that on account of absence from the city I cannot be present at the meeting to-day. I consider it of vital importance that the County Medical Society should declare to-day, in no doubtful manner, that it sustains the honor, the dignity and the honesty of the medical profession; that it repudiates the late action of the State Medical Society, in doing away with the old Code of Ethics and substituting therefor a weak and ridiculous one, virtually acknowledging all the isms and false systems of the day. This action has already done much harm. Let us do all we can to prevent further mischief. T. F. ROCHESTER.

A motion made by Dr. E. Storck to amend Dr. Davidson's resolution by striking out all that which pertained to the instruction of delegates finally prevailed. Dr. Davidson then said that his resolutions, thus amended, did not materially differ in their effect from the original substitute offered by Dr. Gay and he therefore desired to withdraw them.

A vote was then taken on Dr. Gay's substitute for Dr. Hopkins' resolution, which was passed without dissent.

Dr. Matthew D. Mann, Librarian of the Buffalo Medical Library, in behalf of the trustees, asked that the library of the society be entrusted to their keeping. He stated that it would be placed in a more convenient location than at present, and every facility of access to its books would be offered to members of the society by the association. The request was granted.

The following officers were elected for the ensuing year: President, Dr. S. E. S. H. Nott, Hamburg; Vice-President, Dr. Henry R. Hopkins, Buffalo; Secretary, Dr. Arthur M. Barker, Buffalo; Treasurer, Dr. Frank W. Abbott, Buffalo; Librarian, Dr. John B. Samo, Buffalo.

Committee on Membership, Drs. E. T. Dorland, H. Mynter and T. M. Johnson.

Board of Censors—Examiner in Anatomy, Physiology and Surgery, Dr. E. Storck; Examiner in Practice of Obstetrics, Dr. M. D. Mann; Examiner in Materia Medica and Botany, Dr. A. H. Briggs; Examiner in Chemistry and Pharmacy, Dr. P. W. Van Peyma; Examiner in Medical Jurisprudence and Pathology, Dr. F. F. Hoyer.

Dr. Nott, the newly elected President, was conducted to the Chair, and the retiring President, Dr. T. M. Johnson, presented an address on "Pharmacy and Medicine." It had reference to the law introduced in the legislature, concerning the requirements of pharmacists. He urged the passage of the law and a rigid enforcement of its provisions.

A vote of thanks was tendered the Doctor for his able address, and a committee of three appointed, with Dr. Johnson as chairman, to consider the best method of carrying out the plans suggested in the paper.

